# Rest‐Activity Rhythms and Cognition in Older Adults With and Without Insomnia

**DOI:** 10.1111/jsr.70175

**Published:** 2025-08-21

**Authors:** Miranda G. Chappel‐Farley, Zhiwei Zhao, Christine W. Johnston, Shuo Chen, Avelino C. Verceles, Valerie E. Rogers, Daniel J. Buysse, Emerson M. Wickwire, Kristine A. Wilckens

**Affiliations:** ^1^ Department of Psychiatry University of Pittsburgh School of Medicine Pittsburgh Pennsylvania USA; ^2^ Department of Mathematics University of Maryland College Park College Park Maryland USA; ^3^ Division of Pulmonary, Critical Care, and Sleep Medicine, Department of Medicine University of Maryland Baltimore, School of Medicine Baltimore Maryland USA; ^4^ Division of Biostatistics and Bioinformatics, Department of Epidemiology and Public Health University of Maryland Baltimore, School of Medicine Baltimore Maryland USA; ^5^ Retired USA; ^6^ Department of Psychiatry University of Maryland Baltimore, School of Medicine Baltimore Maryland USA

**Keywords:** aging, attention, cognition, insomnia, rest‐activity rhythms, sleep

## Abstract

Insomnia is associated with risk for cognitive deficits. However, the literature assessing cognitive impairments in insomnia is replete with conflicting findings; it is unclear whether individuals with insomnia exhibit impaired cognition or whether specific sleep features consistently predict cognitive performance in insomnia. Disturbance in rest‐activity rhythms may be more directly associated with cognitive deficits in insomnia. In a sample of older adults with (*n* = 30) and without insomnia (*n* = 33), we examined (1) whether insomnia diagnosis was associated with differences in rest‐activity rhythms and cognition, and (2) whether rest‐activity rhythms were associated with cognition across domains. We used a remote comprehensive cognitive battery to test four domains of cognition: attention, inhibition, cognitive flexibility, and episodic memory. Compared to older adults without insomnia, older adults with insomnia exhibited attenuated rest‐activity rhythms, indicated by lower relative amplitude (*F*_1,59_ = 6.96, *p* = 0.01) with greater activity during the rest period (*F*_1,59_ = 7.96, *p* = 0.01). No group differences were found in cognition. Better attention performance was associated with greater amplitude (relative amplitude: *β* = −0.38, *p* = 0.02; amplitude: *β* = −0.45, *p* = 0.01), activity (M10: *β* = −0.38, *p* = 0.01) and less fragmentation of rest‐activity rhythms (intradaily variability: *β* = 0.34, *p* = 0.03), irrespective of insomnia diagnosis. No other cognitive domains were associated with rest‐activity rhythms. Future studies should develop and test interventions to improve rest‐activity rhythms and cognitive outcomes in older adults with and without insomnia.

## 1 Introduction

Insomnia is characterised by difficulty initiating and maintaining sleep and is associated with impairment in daytime functioning and risk for neurodegenerative disease (Shi et al. 2018). However, the literature assessing whether insomnia is associated with impaired cognition is rife with conflicting findings. Some studies suggest insomnia is associated with cognitive impairment (Wardle‐Pinkston et al. 2019), while others show no deficits (Brownlow et al. 2020), deficits in only a subset of patients (Fortier‐Brochu and Morin 2014), or deficits in only select cognitive domains like memory or attention (Fortier‐Brochu and Morin 2014). Recently, 24‐h rest‐activity rhythms (RARs), which are considered a behavioural correlate of circadian rhythms, have emerged as an important biobehavioural predictor of cognitive function in older adults (Zhang et al. 2025). RARs measure the robustness of the sleep–wake cycle by characterising patterns of physical activity and sleep. RARs may be particularly relevant for cognition in older adults with insomnia, given known age‐ (Duffy et al. 2015) and insomnia‐related disruptions to circadian rhythms (Morris et al. 1990). Moreover, insufficient physical activity (Chappel‐Farley et al. 2022) and attenuated RARs confer risk for future cognitive impairment and neurodegenerative disease (Smagula et al. 2019).

The current study aimed to examine whether older adults with and without insomnia exhibited (1) differences in RAR parameters and cognitive domains of attention, inhibition, cognitive flexibility, and episodic memory, and (2) whether RAR parameters were associated with cognitive domains across the full sample. It was hypothesised that RARs would be less robust in older adults with insomnia relative to those without. Specifically, we hypothesised that older adults with insomnia would exhibit lower relative amplitude with greater rhythm fragmentation and activity during the rest period. In line with the view that disrupted RARs may drive associations between insomnia and cognition, we also hypothesised that less robust RARs (i.e., lower relative amplitude and interdaily stability, and greater intradaily variability) would be associated with worse cognitive performance across domains in older adults with insomnia.

## 2 Methods

### 2.1 Participants

Participants were older adults aged 60–85 years with insomnia (*n* = 30) and without insomnia (*n* = 33) recruited from the University of Maryland, Baltimore volunteer registry and surrounding community. All participants provided informed consent in compliance with the University of Maryland, Baltimore Internal Review Board (HP‐00092562). Insomnia diagnosis was defined by (a) meeting DSM‐5 diagnostic criteria for insomnia disorder based on a clinical interview with a board‐certified sleep medicine physician (ACV) or board‐certified sleep psychologist (EMW), and (b) self‐reported moderate or severe insomnia symptoms measured by the Insomnia Severity Index (i.e., ISI ≥ 15 to ensure clinically significant insomnia symptoms) (Bastien et al. 2001) which was completed during the baseline assessment. Control participants did not meet the diagnostic criteria for insomnia and did not report clinically significant insomnia (i.e., ISI < 8). Participants wore an actigraph and completed daily sleep diaries during the 14‐day remote monitoring period, but these data were not used to confirm the insomnia diagnosis, as this has been established elsewhere (Wickwire et al. 2023).

Inclusion criteria included ownership of a smart phone and an internet‐connected home computer to complete remote assessments. Specific inclusion criteria for the insomnia group included: self‐reported sleep duration < 6.5 h, self‐reported daytime impairment, and symptom duration > 6 months. Specific inclusion criteria for the control group included: self‐reported sleep duration ≥ 6.5 h and no self‐reported daytime impairment. Exclusion criteria were based on a telephone interview clinical judgement and included self‐reported diagnosis of severe depression, untreated bipolar disorder or seizures; severe anxiety disorder as determined by General Anxiety Disorder‐7 score ≥ 15; self‐reported uncontrolled medical or psychiatric illness; history of neurological disorder such as stroke or Parkinson's Disease; active treatment for alcohol or substance abuse; attempted suicide within the past 5 years; body mass index ≥ 35, self‐reported use of sedative hypnotics; current tobacco use; unwillingness or inability to wear an actigraphy device. Participants were not excluded for any other sleep or cognitive disorders.

### 2.2 Procedures

As described elsewhere (Wickwire et al. 2023), all study procedures were conducted remotely. All participants completed home sleep apnea testing (HSAT) which was performed using a flow‐based device (Alice NightOne, Philips Respironics) that measured body position, pressure flow, snoring, respiratory effort, blood oxygen level, plethysmography, and heart rate. Participants received verbal and written instructions on HSAT procedures. Devices were mailed to participants and then returned via a third‐party company (MedBridge) that also provided 24‐h support. Raw data were reviewed and interpreted by a board‐certified sleep medicine physician (ACV) based on Medicare criteria. A total of 10 participants had missing HSAT data due to device failure or improper device application (see Table 1). Participants completed the Brief Test of Adult Cognition by Telephone (BTACT) and computerized cognitive testing at home at baseline. Wrist‐worn actigraphy (Actiwatch Spectrum, Philips) was then monitored over 2 weeks to evaluate RARs. Participants were instructed to wear the actigraph continuously during the 14‐day monitoring period, except when bathing or swimming.

**TABLE 1:** Participant demographics, cognitive performance, and RAR measures as a function of insomnia status.

	Control (*n* = 33)	Insomnia (*n* = 30)	Test Statistic	Total *N*	*p*
Age	70.5 ± 5.7 [61, 83]	68.0 ± 6.6 [61, 76]	*t =* 1.61^b^	63	0.11
Female sex	21 (64%)	20 (67%)	*Χ* ^*1*^ = 0.06^a^	63	0.80
Race					
Native American	0	2 (7%)	*Χ* ^*3*^ = 3.49^a^	63	0.32
Black	8 (24%)	4 (17%)			
White	25 (76%)	24 (80%)			
Education					
No College Degree	10 (30%)	6 (20%)	*Χ* ^*5*^ = 3.25^a^	63	0.66
Higher than a College Degree	23 (70%)	24 (80%)			
Apnea Hypopnea Index	6.9 ± 6.6 [1.2, 31.7]	12.1 ± 8.7 [0.6, 35.3]	*t =* −2.54^b^	53	0.01*
Cognitive domain					
Attention	−0.21 ± 0.60 [−1, 1]	0.23 ± 1.27 [−1, 4]	*F*_1,43_ = 0.693^c^	47	0.41
Inhibition	−0.001 ± 0.52 [−0.9, 0.6]	0.02 ± 0.55 [−1.1, 0.8]	*F*_1,59_ = 0.12^c^	63	0.72
Cognitive Flexibility	0.06 ± 0.59 [−1.2, 1.7]	−0.05 ± 0.80 [−1.2, 0.9]	*F*_1,59_ = 0.04^c^	63	0.84
Verbal Episodic Memory	0.13 ± 0.96 [−1.8, 2.1]	−0.14 ± 0.93 [−1.6, 2.2]	*F*_1,59_ = 1.53^c^	63	0.22
RAR					
Non‐parametric					
Interdaily Stability (IS)	0.59 ± 0.11 [0.4, 0.8]	0.53 ± 0.14 [0.2, 0.7]	*F*_1,59_ = 3.47^c^	63	0.07^c^
Intradaily Variability (IV)	0.39 ± 0.13 [0.2, 0.7]	0.44 ± 0.14 [0.3, 0.8]	*F*_1,59_ = 2.37^c^		0.13
M10	3.80 ± 0.56 [2.8, 4.9]	3.82 ± 0.56 [2.4, 5.0]	*F*_1,59_ = 0.02^c^		0.90
L5	0.40 ± 0.17 [0.2, 0.9]	0.58 ± 0.36 [0.01, 1.5]	*F*_1,59_ = 7.96^c^		0.01*
Parametric Cosinor					
Relative Amplitude (RA)	0.81 ± 0.08 [0.5, 0.9]	0.75 ± 0.13 [0.4, 1.0]	*F*_1,59_ = 6.96^c^	63	0.01*
Amplitude	1.75 ± 0.34 [1.1, 2.5]	1.63 ± 0.43 [0.6, 2.3]	*F*_1,59_ = 2.22^c^		0.14
Acrophase	14.56 ± 1.26 [11.5, 17.3]	15.13 ± 1.39 [11.2, 17.4]	*F*_1,59_ = 2.92^c^		0.09**
MESOR	2.45 ± 0.40 [1.9, 3.3]	2.52 ± 0.44 [1.3, 3.3]	*F*_1,59_ = 0.45^c^		0.51

*Note*: x ± s represents mean ± 1 SD. N is the number of non‐missing values. Minimum and maximum values are displayed in brackets for continuous variables. *p < 0.05, **p < 0.10.

^a^ chi square test was performed for this comparison.

^b^ t‐test was performed for this comparison.

^c^ ANCOVA adjusting for age and sex.

### 2.3 Rest‐Activity Rhythm Parameters

#### 2.3.1 Actigraphy Data Preprocessing

Actigraphy data were preprocessed following the procedures outlined by Alfini et al. (2021) using R software. One participant was excluded due to an insufficient number (two 24‐h periods) of 24‐h periods with complete actigraphy data. Average wear time for the entire sample was 11.94 ± 2.29 days. Twenty‐four hour periods with > 5% missing data were excluded. Mean values were determined for consecutive 30‐s epochs and then log‐transformed, resulting in within‐subject minute‐level data. Missing data were imputed with average minute‐level values from the same time of day measured on all other days during the study period. Minute‐level data were aggregated into 144 10‐min bins per 24‐h period for each participant.

#### 2.3.2 Data Reduction for RAR Calculations

To determine RAR parameters, 24‐h periods were aligned to begin at midnight. Next, standard non‐parametric RARs were generated using the ActCR package in R: intradaily variability (IV), interdaily stability (IS), relative amplitude (RA), average activity over the 10‐h activity maximum (M10), level of activity over the 5‐h activity minimum (L5). Supplementary standard parametric cosinor RARs were also generated with the cosinor R package: amplitude, acrophase, and midline estimating statistic of rhythm (MESOR). See Table 2 for definitions of each RAR measure.

**TABLE 2:** Regression models predicting cognitive performance from rest‐activity rhythms controlling for age, sex, and insomnia status.

RAR parameter	Description	Attention	Inhibition	Flexibility	Verbal memory
Interdaily stability (IS)	Consistency of rest‐activity rhythm across 24‐h periods	*β* = −0.19, *t* = −1.15, *p* = 0.26, *n* = 47	*β* = 0.02, *t* = 0.16, *p* = 0.88, *n* = 63	*β* = −0.11, *t* = −0.78, *p* = 0.44, *n* = 63	*β* = 0.07, *t* = 0.53, *p* = 0.60, *n* = 63
Intradaily variability (IV)	Fragmentation of 24‐h rest‐activity rhythm	***β* = 0.34,** ***t* = 2.26,** *p* = 0.03*, ***n* = 47**	*β* = −0.08, *t* = −0.54, *p* = 0.59, *n* = 63	*β* = −0.09, *t* = −0.64, *p* = 0.52, *n* = 63	*β* = −0.06, *t* = −0.47, *p* = 0.64, *n* = 63
Relative amplitude (RA)	Robustness of rest‐activity rhythm calculated as the relative difference between active and rest periods (normalized difference between most active 10‐h and least active 5‐h)	***β* = −0.38,** ***t* = −2.37,** ***p* = 0.02^t^,** *n* = 47	*β* = 0.03, *t* = 0.20, *p* = 0.84, *n* = 63	*β* = 0.03, *t* = 0.24, *p* = 0.81, *n* = 63	*β* = 0.10, *t* = 0.73, *p* = 0.47, *n* = 63
Amplitude	Amplitude of active period	***β* = −0.45,** ***t* = −2.79,** *p* = 0.01*, *n* = 47	*β* = 0.20, *t* = 1.38, *p* = 0.17, *n* = 63	*β* = 0.04, *t* = 0.29, *p* = 0.77, *n* = 63	*β* = 0.10, *t* = 0.73, *p* = 0.47, *n* = 63
Acrophase	24‐h clock time of peak activity	*β* = 0.02, *t* = 0.11, *p* = 0.91, *n* = 47	*β* = −0.02, *t* = −0.15, *p* = 0.88, *n* = 63	*β* = 0.04, *t* = 0.31, *p* = 0.76, *n* = 63	*β* = 0.13, *t* = 0.11, *p* = 0.29, *n* = 63
MESOR	Mean activity level across 24‐h periods	*β* = −0.27, *t* = −1.85, *p* = 0.07^t^, *n* = 47	*β* = 0.14, *t* = 1.05, *p* = 0.29, *n* = 63	*β* = 0.09, *t* = 0.70, *p* = 0.49, *n* = 63	*β* = −0.4, *t* = −0.29, *p* = 0.77, *n* = 63
M10	Period of maximum activity over 10 consecutive hours	***β* = −0.38,** ***t* = −2.56,** *p* = 0.01*, *n* = 47	*β* = 0.19, *t* = 1.42, *p* = 0.16, *n* = 63	*β* = 0.10, *t* = 0.77, *p* = 0.45, *n* = 63	*β* = 0.01, *t* = 0.08, *p* = 0.94, *n* = 63
L5	Period of minimum activity over 5 consecutive hours	*β* = 0.25, *t* = 1.56, *p* = 0.13, *n* = 47	*β* = 0.03, *t* = 0.22, *p* = 0.82, *n* = 63	*β* = −0.01, *t* = −0.07, *p* = 0.95, *n* = 63	*β* = −0.08, *t* = −0.60, *p* = 0.55, *n* = 63

*Note*: Standardised beta coefficients are presented with corresponding *t* and *p* value. *indicates *p* < 0.05 and ^t^indicates *p* < 0.10 after additionally adjusting for education. The bold text was used to signify statistical significance between groups. However, the * indicator is sufficient.

### 2.4 Cognitive Testing

As described elsewhere (Wickwire et al. 2023), all assessments and procedures for this study were administered remotely, including cognitive assessments. This remote testing approach was selected partly due to COVID‐19‐related restrictions at the time the study began, to limit participant burden, and to assess the feasibility of obtaining quality data with remote assessments (Wickwire et al. 2023).

Participants completed the remote cognitive testing on their own personal computers. First, the study coordinator instructed participants how to download the link to remote cognitive tasks and how to open them on their home computer using EPrime Go. The study coordinator then remained on the phone with participants for the duration of the cognitive testing to provide technical support if needed. Participants completed three computerized cognitive tasks remotely in the following order: the psychomotor vigilance task (PVT) to assess attention, the Stroop Task to measure inhibition, and a task‐switching paradigm to assess cognitive flexibility. Remote cognitive testing took 30‐minutes to complete. These tasks were specifically selected as they can be easily completed remotely and have been shown to be highly sensitive to individual differences in sleep in older adults (Wilckens et al. 2017; Blatter et al. 2006). A total of 16 participants (*n* = 8 per group) were unable to complete the PVT due to technical issues. Participants completed the BTACT (Lachman et al. 2014) on a separate day. The BTACT was selected as it allows for a brief assessment of neuropsychological performance using a standard battery of conventional tasks to assess episodic memory and executive function. Composite scores of attention, inhibition, cognitive flexibility, and verbal episodic memory were derived from the outcome measures of the BTACT and the computerised tests by averaging the z‐scores for each outcome of interest within each a priori defined domain (see Table 3 for details).

**TABLE 3:** Task components comprising each cognitive composite score.

Domain	Measures	Score
Attention	PVT	Lapses (RT > 500 ms) Median RT
Inhibition	Stroop	RT for incongruent trials
	Stroop	Accuracy for incongruent trials
	Stroop	((RT for incongruent trials − RT for congruent trials)/RT for congruent trials) * 100
	BTACT	Performance on inhibition trials (red‐green switch)
Cognitive flexibility	Task‐switching paradigm: Global Switching Costs in RT	Repeat Trial RT − Single Task RT
	Task‐switching paradigm: Global Switching Costs in Accuracy	Single Task Accuracy − Repeat Trial Accuracy
	BTACT	Flexibility: proportion of correct switching responses
Verbal memory	BTACT	Immediate test word recall score
	BTACT	Total word recall score (immediate + delayed)

Abbreviations: BTACT, Brief Test of Adult Cognition by Telephone; PVT, psychomotor vigilance task; RT, reaction time; Stroop, Stroop Task.

### 2.5 Statistical Analyses

Statistical analyses were performed with IBM SPSS 29 and RStudio 2022.12.0 + 353. See Table 1 for between group differences in participant characteristics, RAR measures, and cognitive composites. Group comparisons in demographic variables were examined using chi‐square tests for categorical variables and independent samples t‐tests for continuous variables. Group differences in RAR measures and cognitive composites were examined using ANCOVA models adjusting for age and sex. The attention score, RA, L5, and the Apnea‐Hypopnea Index (AHI)‐a measure of obstructive sleep apnea (OSA) severity‐were log‐transformed, and a constant was added to meet normality assumptions.

First, multiple regression models tested for an interaction between RARs and insomnia status predicting cognitive composite scores, adjusting for age and sex as covariates. Then, main effects were examined using multiple regression models testing associations between RARs and cognitive composites across the entire sample, adjusting for age, sex, and insomnia status as covariates (Table 3). Sensitivity analyses included either education or AHI as an additional covariate. Using R software, Benjamini‐Hochberg False Discovery Rate (FDR) correction was applied to regression models predicting each cognitive composite (i.e., 8 total comparisons for each composite due to 8 RAR variables). Results are presented before and after correcting for multiple comparisons. Additional subgroup analyses controlling for covariates were performed to determine if findings were driven by insomnia status.

## 3 Results

### 3.1 Participants

The final sample included 30 older adults with insomnia and 33 older adults without insomnia (total sample *μ*_age_ = 69.2 ± 6.2). No baseline differences were observed in demographic characteristics aside from AHI (Table 1). A total of 19 older adults with insomnia also met clinical criteria for OSA (*n* = 19 > mild OSA), and 14 older adults without insomnia met OSA criteria (*n* = 14 > mild OSA).

### 3.2 Between‐Group Differences in RAR Parameters and Cognition

As presented in Table 1, relative to older adults without insomnia, those with insomnia exhibited significantly lower relative amplitude (RA, *F*_1,59_ = 6.96, *p* = 0.01, *η* ^2^ = 0.11) and higher minimum 5‐h period activity level (L5, *F*_1,59_ = 7.96, *p* = 0.01, *η* ^2^ = 0.12), indicating more activity during their least active 5‐h period. The insomnia group also exhibited greater amounts of activity later in the day with less consistent rhythms, as evidenced by marginal differences in acrophase (*F*_1,59_ = 2.92, *p* = 0.09, *η* ^2^ = 0.05) and interdaily stability (IS, *F*_1,59_ = 3.47, *p* = 0.07, *η* ^2^ = 0.06). No group differences were observed across cognitive domains.

### 3.3 Associations Between RAR Parameters and Cognition

**FIGURE 1:**
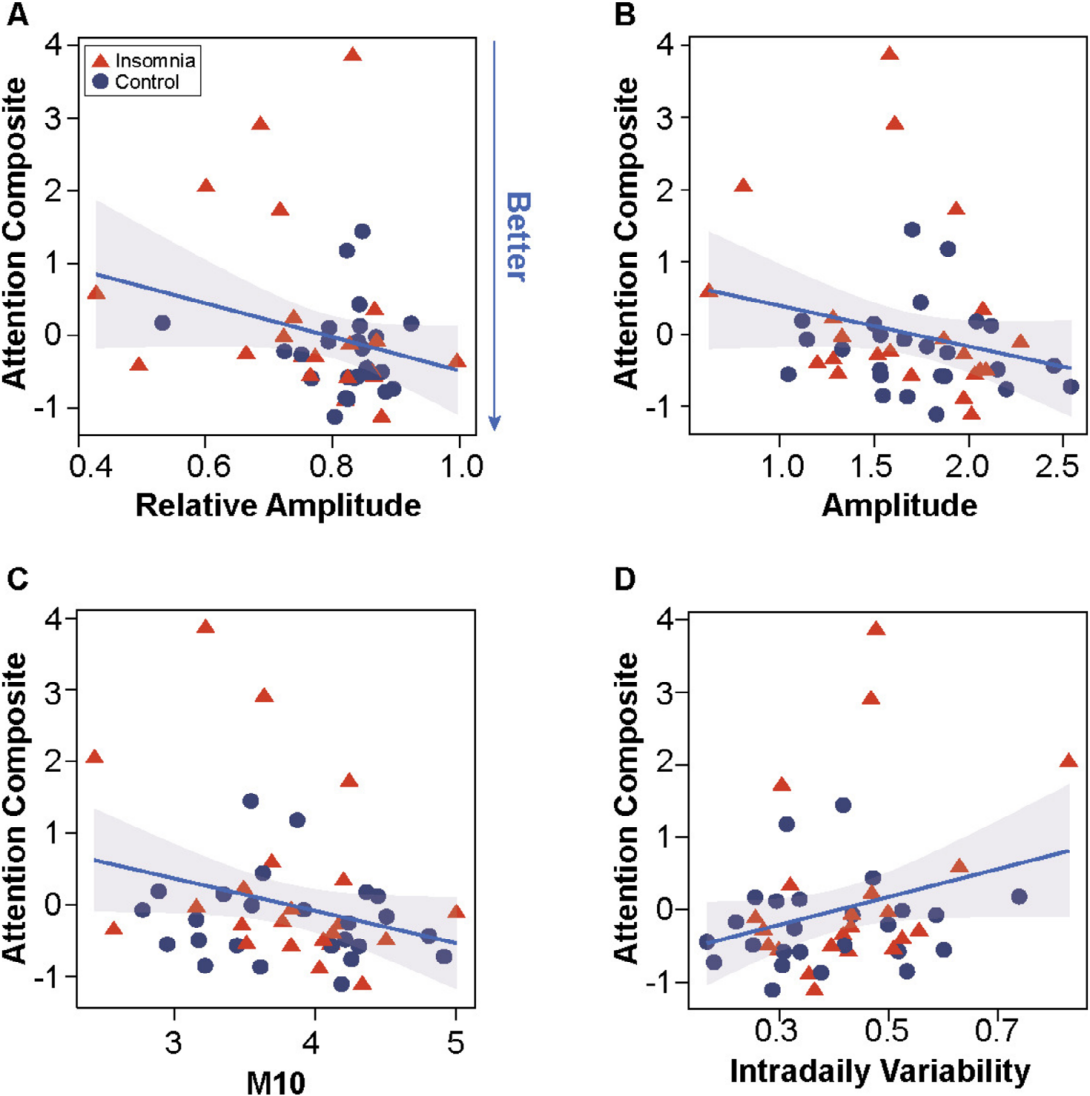
More robust and stable rest‐activity rhythms are associated with better attention performance in older adults with and without insomnia. Scatterplots showing the correlations between RAR variables and the attention composite score across the entire sample show that greater (A) relative amplitude, (B) amplitude, and (C) M10 are associated with lower attention composite scores, indicating better performance. (D) Greater intradaily variability is associated with a higher attention composite score.

No significant interactions were observed between insomnia status and any RAR parameter when predicting each of the cognitive composite scores (Table S1). Thus, we tested the main effects of each RAR measure on each cognitive outcome across all participants (Table 3). Among RAR measures, higher RA (*β* = −0.38, *t* = −2.37, *p* = 0.02, FDR‐adjusted *p* = 0.05; Figure 1A), amplitude (*β* = −0.45, *t* = −2.79, *p* = 0.01, FDR‐adjusted *p* = 0.04; Figure 1B), M10 (*β* = −0.38, *t* = −2.56, *p* = 0.01, FDR‐adjusted *p* = 0.04; Figure 1C), and lower intradaily variability (IV, *β* = 0.34, *t* = 2.26, *p* = 0.03, FDR‐adjusted *p* = 0.06; Figure 1D) were associated with lower attention composite scores (i.e., better performance). Sensitivity analyses including AHI as an additional covariate (*n* = 41) produced similar results (RA: *β* = −0.46, *t* = −2.65, *p* = 0.01; amplitude: *β* = −0.35, *t* = −2.08, *p* = 0.05; IV: *β* = 0.29, *t* = 1.82, *p* = 0.08). Similar sensitivity analyses instead adjusting for education found significant associations with amplitude (*β* = −0.40, *t* = −2.52, *p* = 0.02) and M10 (*β* = −0.36, *t* = −2.44, *p* = 0.02). See Table S2 for regression results stratified by insomnia status. These stratified models suggest that these relationships are primarily driven by the insomnia group, as greater amplitude (*β* = −0.53, *t* = −2.19, *p* = 0.04, *n* = 22) and M10 (*β* = −0.45, *t* = −2.07, *p* = 0.05, *n* = 22) were significantly associated with better attention performance only in the insomnia group, while adjusting for age and sex. Similarly, greater intradaily variability (*β* = 0.45, *t* = 1.88, *p* = 0.08, *n* = 22) and relative amplitude (*β* = −0.46, *t* = −2.03, *p* = 0.06, *n*=22) exhibited trending relationships with attention in older adults with insomnia.

## 4 Discussion

Relative to older adults without insomnia, those with insomnia demonstrated less robust RARs, driven by greater activity during the rest period, a time when activity is normally minimal. Although no between‐group differences were detected in cognition, several metrics of stronger and more stable RARs were associated with better attention performance. These findings underscore the importance of considering circadian influences, including RARs, in understanding and treating cognitive deficits in older adults.

Relative to older adults without insomnia, those with insomnia exhibited lower RAR amplitude and a greater level of activity over the 5‐h activity minimum (i.e., rest period), rather than distinct differences in activity between day and night that reflect robust RARs. These findings are consistent with the literature (Spira et al. 2015; Pollak et al. 1992). Increased hyperarousal across the 24‐h period (Riemann et al. 2010) may be an underlying mechanism contributing to dampened rhythms in insomnia, driving greater activity later in the day and more activity during the rest period, as observed in the current study. However, no significant interactions between insomnia status and RARs were observed, and stratified analyses revealed that associations were in the same direction for both groups. Nonetheless, the relationships between RARs and attention only reached statistical significance in the insomnia group. Thus, any group differences or lack thereof should be interpreted with caution. Larger studies, including experimental manipulations of various aspects of insomnia symptoms and RARs, are needed to determine whether these factors interact or amplify one another in relation to cognition. Additionally, future work should examine whether behavioral strategies, such as increasing physical activity early in the day, may enhance rhythm robustness and reduce insomnia symptoms by promoting sleep consolidation.

Across the entire sample, RAR measures of amplitude and rhythmicity were associated with better sustained attention, consistent with existing literature (Zhang et al. 2025). Sustained attention varies with the circadian rhythm and is particularly sensitive to sleep deprivation, more so than more cognitively demanding executive function tasks (Lim and Dinges 2008). Notably, both unstable RARs (Espinosa et al. 2025; McMahon et al. 2020) and insufficient sleep (Lim and Dinges 2008) have been linked to changes in frontal and parietal networks associated with sustained attention. The current results may similarly support specificity of the relationship between RARs and attention rather than domain‐general associations with executive function and memory, perhaps due to shared neural underpinnings. Nonetheless, the study design may have contributed to the specific associations of RARs with attention. For instance, participants self‐selected the time of remote cognitive testing, which proceeded in the order of PVT first, followed by the Stroop Task and Task‐Switch. It is possible that participants self‐selected cognitive testing times in line with their circadian preference, which may have bolstered initial performance on the PVT, contributing to relationships with RARs. Future studies are needed to address whether sustained attention is indeed more sensitive to RARs with investigations of underlying neurobiological mechanisms and manipulations of time‐on‐tasks and time of day.

The current study suggests that individual differences in circadian rhythmicity—inferred by RAR parameters—might be a key biobehavioral factor underlying cognitive deficits in insomnia. As a majority of prior studies in the insomnia‐cognition literature focus primarily on sleep metrics and rarely assess circadian functioning, this methodological gap may partially explain the inconsistent findings. Therefore, it will be important for future studies with larger samples to examine RARs as a mediator of cognitive impairments in insomnia. Altogether, these data suggest that attenuated RARs may contribute to cognitive deficits in older adults with and without insomnia. These results advance our understanding of biobehavioral mechanisms supporting cognition in older adults, highlighting the importance of the dynamic interplay of circadian factors, physical activity, and sleep in cognitive function.

## 5 Limitations and Future Directions

Comprehensive remote assessments of RARs and cognition across multiple domains are major strengths of this study. However, our sample size was relatively small and highly educated, potentially limiting generalizability. Even so, we found moderate‐to‐large effects that provide preliminary evidence that RARs may be an important determinant of cognition in older adults with insomnia for future studies with larger samples. Of note, several participants with insomnia also exhibited comorbid OSA, though this was adjusted for in sensitivity analyses. As research suggests that OSA impacts circadian rhythmicity (Soreca 2021), physical activity engagement, sleep quality, and cognitive function (Chappel‐Farley et al. 2022), it will be important for future investigations to examine RAR‐cognition relationships across sleep disorder phenotypes, specifically in older adults with isolated insomnia, OSA, or both. Similarly, prospective studies should examine whether improvements in RARs predict cognitive gains from sleep disorder treatment. While we excluded participants based on uncontrolled major psychiatric or medical disorder, we were unable to assess the effects of medication usage or other underlying medical comorbidities including cognitive impairment on the association between RARs and cognition. Despite these limitations, our findings provide greater insight into the mechanisms underlying the heterogeneity in insomnia‐cognition literature; whether individuals have a distinct active and sleep period may be a determinant of whether cognitive impairments arise.

In this study, older adults with insomnia exhibited attenuated RARs driven by greater activity during the rest period. More robust RARs with greater daily amplitude were associated with better attention in older adults, irrespective of insomnia diagnosis. Whether interventions to improve RARs improve cognition in older adults with and without insomnia will be an important avenue for future investigation.

## Author Contributions

**Miranda G. Chappel‐Farley:** formal analysis, visualization, writing – original draft, writing – review and editing. **Zhiwei Zhao:** data curation, formal analysis, visualization, writing – review and editing. **Christine W. Johnston:** conceptualization, data curation, investigation, resources, project administration, supervision, writing – review and editing. **Shuo Chen:** formal analysis, supervision, writing – review and editing. **Avelino C. Verceles:** data curation, supervision, writing – review and editing. **Valerie E. Rogers:** methodology, writing – review and editing. **Daniel J. Buysse:** conceptualization, writing – review and editing. **Emerson M. Wickwire:** conceptualization, funding acquisition, resources, supervision, writing – review and editing. **Kristine A. Wilckens:** conceptualization, methodology, supervision, validation, writing – original draft, writing – review and editing.

## Conflicts of Interest

Dr. Wickwire's institution has received research funding from the American Academy of Sleep Medicine Foundation, Department of Defense, Merck, National Institutes of Health/National Institute on Agin, ResMed, the ResMed Foundation, and the Sleep Research Society Foundation. Dr. Wickwire has served as a scientific consultant to Axsome Therapeutics, DayZz, Eisai, EnsoData, Idorsia, Merck, Nox Health, Primasun, Purdue, and ResMed and is an equity shareholder in WellTap. Dr. Chappel‐Farley has served as a consultant to Apnimed Inc, Dr. Buysse has served as a consultant to Sleep Number.

## Supporting information


**Table S1:** Regression models testing interactions between RAR parameters and insomnia status predicting each cognitive composite score. All models adjusted for age and sex.
**Table S2:** Regression models testing the association between RAR parameters and cognitive performance stratified by insomnia status. All models adjusted for age and sex.

## Data Availability

The data that support the findings of this study are available upon reasonable request from Emerson Wickwire, PhD at ewickwire@som.umaryland.edu.

## References

[jsr70175-bib-0001] Alfini, A. , M. Albert , A. V. Faria , et al. 2021. “Associations of Actigraphic Sleep and Circadian Rest/Activity Rhythms With Cognition in the Early Phase of Alzheimer's Disease.” SLEEP Advances 2: zpab007. 34095836 10.1093/sleepadvances/zpab007 PMC8168567

[jsr70175-bib-0002] Bastien, C. H. , A. Vallières , and C. M. Morin . 2001. “Validation of the Insomnia Severity Index as an Outcome Measure for Insomnia Research.” Sleep Medicine 2: 297–307. 11438246 10.1016/s1389-9457(00)00065-4

[jsr70175-bib-0003] Blatter, K. , P. Graw , M. Münch , V. Knoblauch , A. Wirz‐Justice , and C. Cajochen . 2006. “Gender and Age Differences in Psychomotor Vigilance Performance Under Differential Sleep Pressure Conditions.” Behavioural Brain Research 168: 312–317. 16386807 10.1016/j.bbr.2005.11.018

[jsr70175-bib-0004] Brownlow, J. A. , K. E. Miller , and P. R. Gehrman . 2020. “Insomnia and Cognitive Performance.” Sleep Medicine Clinics 15: 71–76. 32005351 10.1016/j.jsmc.2019.10.002 PMC7000136

[jsr70175-bib-0005] Chappel‐Farley, M. G. , B. A. Mander , A. B. Neikrug , et al. 2022. “Symptoms of Obstructive Sleep Apnea Are Associated With Less Frequent Exercise and Worse Subjective Cognitive Function Across Adulthood.” Sleep 45: 1–10. 10.1093/sleep/zsab240 PMC8919199 34604910

[jsr70175-bib-0006] Duffy, J. F. , K.‐M. Zitting , and E. D. Chinoy . 2015. “Aging and Circadian Rhythms.” Sleep Medicine Clinics 10: 423–434. 26568120 10.1016/j.jsmc.2015.08.002 PMC4648699

[jsr70175-bib-0007] Espinosa, N. , C. M. Hoyos , A. C. McKinnon , H. Almgren , S. L. Duffy , and S. L. Naismith . 2025. “Rest‐Activity Rhythm Fragmentation and Synchronization Are Linked With Reduced Cortical Thickness in Older Adults ‘at Risk’ for Dementia.” Sleep 48: zsaf017. 40052961 10.1093/sleep/zsaf017 PMC12068054

[jsr70175-bib-0008] Fortier‐Brochu, É. , and C. M. Morin . 2014. “Cognitive Impairment in Individuals With Insomnia: Clinical Significance and Correlates.” Sleep 37: 1787–1798. 25364074 10.5665/sleep.4172 PMC4196062

[jsr70175-bib-0009] Lachman, M. E. , S. Agrigoroaei , P. A. Tun , and S. L. Weaver . 2014. “Monitoring Cognitive Functioning: Psychometric Properties of the Brief Test of Adult Cognition by Telephone.” Assessment 21: 404–417. 24322011 10.1177/1073191113508807 PMC4050038

[jsr70175-bib-0010] Lim, J. , and D. F. Dinges . 2008. “Sleep Deprivation and Vigilant Attention.” Annals of the New York Academy of Sciences 1129: 305–322. 18591490 10.1196/annals.1417.002

[jsr70175-bib-0011] McMahon, M. , Y. Malneedi , D. A. Worthy , and D. M. Schnyer . 2020. “Rest‐Activity Rhythms and White Matter Microstructure Across the Lifespan.” Sleep 44: zsaa266. 10.1093/sleep/zsaa266 PMC8193551 33269397

[jsr70175-bib-0012] Morris, M. , L. Lack , and D. Dawson . 1990. “Sleep‐Onset Insomniacs Have Delayed Temperature Rhythms.” Sleep 13: 1–14. 2305166 10.1093/sleep/13.1.1

[jsr70175-bib-0013] Pollak, C. P. , D. Perlick , and J. P. Linsner . 1992. “Daily Sleep Reports and Circadian Rest‐Activity Cycles of Elderly Community Residents With Insomnia.” Biological Psychiatry 32: 1019–1027. 1467382 10.1016/0006-3223(92)90063-6

[jsr70175-bib-0014] Riemann, D. , K. Spiegelhalder , B. Feige , et al. 2010. “The Hyperarousal Model of Insomnia: A Review of the Concept and Its Evidence.” Sleep Medicine Reviews 14: 19–31. 19481481 10.1016/j.smrv.2009.04.002

[jsr70175-bib-0015] Shi, L. , S. J. Chen , M. Y. Ma , et al. 2018. “Sleep Disturbances Increase the Risk of Dementia: A Systematic Review and Meta‐Analysis.” Sleep Medicine Reviews 40: 4–16. 28890168 10.1016/j.smrv.2017.06.010

[jsr70175-bib-0016] Smagula, S. F. , S. Gujral , C. S. Capps , and R. T. Krafty . 2019. “A Systematic Review of Evidence for a Role of Rest‐Activity Rhythms in Dementia.” Frontiers in Psychiatry 10: 778. 31736798 10.3389/fpsyt.2019.00778 PMC6832024

[jsr70175-bib-0017] Soreca, I. 2021. “The Role of Circadian Rhythms in Obstructive Sleep Apnea Symptoms and Novel Targets for Treatment.” Chronobiology International 38: 1274–1282. 34027758 10.1080/07420528.2021.1929281

[jsr70175-bib-0018] Spira, A. P. , V. T. Runko , P. H. Finan , et al. 2015. “Circadian Rest/Activity Rhythms in Knee Osteoarthritis With Insomnia: A Study of Osteoarthritis Patients and Pain‐Free Controls With Insomnia or Normal Sleep.” Chronobiology International 32: 242–247. 25290041 10.3109/07420528.2014.965314 PMC4394999

[jsr70175-bib-0019] Wardle‐Pinkston, S. , D. C. Slavish , and D. J. Taylor . 2019. “Insomnia and Cognitive Performance: A Systematic Review and Meta‐Analysis.” Sleep Medicine Reviews 48: 101205. 31522135 10.1016/j.smrv.2019.07.008

[jsr70175-bib-0020] Wickwire, E. M. , A. C. Verceles , S. Chen , et al. 2023. “Smart Phone/Ecological Momentary Assessment of Sleep and Daytime Symptoms Among Older Adults With Insomnia.” American Journal of Geriatric Psychiatry 31: 372–378. 10.1016/j.jagp.2023.01.020 36813640

[jsr70175-bib-0021] Wilckens, K. A. , M. H. Hall , K. I. Erickson , et al. 2017. “Task Switching in Older Adults With and Without Insomnia.” Sleep Medicine 30: 113–120. 28215233 10.1016/j.sleep.2016.09.002 PMC5321623

[jsr70175-bib-0022] Zhang, M. , N.‐C. Chi , S. E. Gardner , and C. Moon . 2025. “Rest‐Activity Rhythm and Cognitive Function in Older Adults: A Scoping Review and Integrative Framework.” Geriatric Nursing 61: 80–90. 39546912 10.1016/j.gerinurse.2024.10.074

