# Supplementary material for: Rest‐Activity Rhythms and Cognition in Older Adults With and Without Insomnia
Source: J Sleep Res. 2025 Aug 21;35(2):e70175. doi: 10.1111/jsr.70175 (PMC13003282; doi:10.1111/jsr.70175)
Supplement: Supplementary file 1 — Table S1: Regression models testing interactions between RAR parameters and insomnia status predicting each cognitive composite score. All models adjusted for age and sex. Table S2: Regression models testing the association between RAR parameters and cognitive performance stratified by insomnia status. All models adjusted for age and sex. [file JSR-35-e70175-s001.docx]

***Supplemental Materials for***

**Rest-activity rhythms and cognition in older adults with and without insomnia**

**Miranda G. Chappel-Farley^1^, Zhiwei Zhao^2^, Christine W. Johnston^3^, Shuo Chen^4^, Avelino C. Verceles^3^, Valerie E. Rogers^5^, Daniel J. Buysse^1^, Emerson M. Wickwire^3*^, Kristine A. Wilckens^1*^**

**^1^** Department of Psychiatry, University of Pittsburgh School of Medicine, Pittsburgh, PA

^2^ Department of Mathematics, University of Maryland College Park, College Park, MD

^3^ Division of Pulmonary, Critical Care, and Sleep Medicine, Department of Medicine, University of Maryland School of Medicine, Baltimore, MD

^4^Division of Biostatistics and Bioinformatics, Department of Epidemiology and Public Health, University of Maryland School of Medicine, Baltimore, MD

^5^Retired

*These authors have contributed equally as senior authors.

**Corresponding Author:**

Miranda G. Chappel-Farley, Ph.D | 3811 O’Hara StUPMC Western Psychiatric Hospital, Pittsburgh, PA, 15213 | [chappelfarleymg@upmc.edu](mailto:chappelfarleymg@upmc.edu)

**Table S1.** Regression models testing interactions between RAR parameters and insomnia status predicting each cognitive composite score, adjusting for age and sex.

| **Interaction Term** | **Attention**  (n=47) | **Inhibition**  (n=63) | **Flexibility**  (n=63) | **Verbal Memory** (n=63) |
| --- | --- | --- | --- | --- |
| IS x Insomnia Status | β=-0.17, SE=0.39, p=0.67 | β=-1.87, SE=1.10, p=0.10 | β=-0.76, SE=1.42, p=0.59 | β=-2.70, SE=1.87, p=0.16 |
| IV x Insomnia Status | β=0.49, SE=0.35, p=0.18 | *β=1.86, SE=1.01, p=0.07*^t^ | β=-0.71, SE=1.31, p=0.59 | β=1.85, SE=1.26, p=0.29 |
| RA x Insomnia Status | β=-1.36, SE=2.20, p=0.54, | β=-2.17, SE=6.27, p=0.73 | β=-3.43, SE=7.93, p=0.67 | β=-16.03, SE=10.35, p=0.13 |
| Amplitude x Insomnia Status | β=-0.12, SE=0.12, p=0.31 | β=-0.55, SE=0.36, p=0.14 | β=0.0002, SE=0.47, p=0.99 | β=-0.68, SE=0.62, p=0.28 |
| Acrophase x Insomnia Status | β=-0.04, SE=0.05, p=0.43 | β=-0.06, SE=0.11, p=0.55 | β=-0.05, SE=0.14, p=0.74 | β=-0.03, SE=0.18, p=0.88 |
| MESOR x Insomnia Status | β=-0.07, SE=0.11, p=0.52 | β=-0.46, SE=0.33, p=0.17 | β=0.51, SE=0.42, p=0.23 | β=-0.39, SE=0.57, p=0.50 |
| M10 x Insomnia Status | β=-0.09, SE=0.08, p=0.24 | β=-0.43, SE=0.24, p=0.17 | β=0.32, SE=0.31, p=0.31 | β=-0.39, SE=0.42, p=0.36 |
| L5 x Insomnia Status | β=0.70, SE=0.95, p=0.46 | β=-1.35, SE=2.48, p=0.59, n=63 | β=2.49, SE=3.13, p=0.43 | β=4.23, SE=4.16, p=0.31 |

IS—Interdaily Stability; IV—Intradaily Variability; RA—Relative Amplitude; β—Standardized regression coefficient; **p*<0.05, ^t^*p*<0.10

**Table S2.** Regression models of the association between rest-activity rhythms and cognitive performance from rest-activity rhythms stratified by insomnia status. All models adjusted for age and sex.

| **RAR Parameter** | **Group** | **Attention** | **Inhibition** | **Flexibility** | **Verbal Memory** |
| --- | --- | --- | --- | --- | --- |
| **IS** | Control | β=-0.17,  n=25 | β=0.19,  n=33 | β=-0.43,  n=33 | β=0.23,  n=33 |
|  | Insomnia | β=-0.17,  n=22 | β=-0.12,  n=30 | β=-0.13,  n=30 | β=-0.07,  n=30 |
| **IV** | Control | β=0.23,  n=25 | β=-0.26,  n=33 | β=0.01,  n=33 | β=-0.17,  n=33 |
|  | Insomnia | *β=0.45^t^*  *n=22* | β=0.10,  n=30 | β=-0.20,  n=30 | β=0.04,  n=30 |
| **RA** | Control | β=-0.27,  n=25 | β=0.08,  n=33 | β=0.07,  n=33 | β=0.30,  n=33 |
|  | Insomnia | *β=-0.46^t^*  *n=22* | β=0.15,  n=30 | β=0.02,  n=33 | β=0.004,  n=30 |
| **Amplitude** | Control | β=-0.33,  n=25 | β=0.29,  n=33 | β=0.01,  n=33 | β=0.21,  n=33 |
|  | Insomnia | **β=-0.53*,**  **n=22** | β=0.14,  n=30 | β=0.09,  n=30 | β=0.02,  n=30 |
| **Acrophase** | Control | β=0.15,  n=25 | β=0.18,  n=33 | β=0.12,  n=33 | β=0.17,  n=33 |
|  | Insomnia | β=-0.08,  n=25 | β=-0.09,  n=30 | β=-0.003,  n=30 | β=0.12,  n=30 |
| **MESOR** | Control | β=-0.23,  n=25 | β=0.25,  n=33 | β=-0.10,  n=33 | β=0.03,  n=33 |
|  | Insomnia | β=-0.30,  n=22 | β=-0.04,  n=30 | β=0.23,  n=30 | β=-0.12,  n=30 |
| **M10** | Control | β=-0.31,  n=25 | β=0.29,  n=33 | β=-0.66,  n=33 | β=0.10,  n=33 |
|  | Insomnia | **β=-0.45*,**  **n=22** | β=0.04,  n=30 | β=0.24,  n=30 | β=-0.09,  n=30 |
| **L5** | Control | β=0.10,  n=25 | β=0.93,  n=33 | β=-0.10,  n=33 | β=-0.21,  n=33 |
|  | Insomnia | β=0.35,  n=22 | β=-0.16,  n=30 | β=0.05,  n=30 | β=-0.04,  n=30 |

IS—Interdaily Stability; IV—Intradaily Variability; RA—Relative Amplitude; β—Standardized regression coefficient; **p*<0.05, ^t^*p*<0.10
